# Pollinator activity and pollination success of *Medicago sativa* L. in a natural and a managed population

**DOI:** 10.1002/ece3.4256

**Published:** 2018-08-19

**Authors:** Min Chen, Xue‐Yong Zhao, Xiao‐An Zuo

**Affiliations:** ^1^ Northwest Institute of Eco‐Environment and Resources CAS Lanzhou China

**Keywords:** breeding system, pollination, pollinator activity, pollinator visitation rate, seed set

## Abstract

*Medicago sativa* L. is an important cash crop in the arid region of northwest China. Pollinator activity is an essential aspect of pollination success, but the relationships between pollinator visitation rate and seed set still need further study of *M. sativa*. We investigated the following characteristics of *M. sativa* in natural and managed populations: floral traits, pollinator activity, and breeding system. Our results indicated the management could affect the number of flowers produced; however, there was no detectable effect on the seed set per flower. We found the percentage of seeds among pollinated flowers in the managed population was significantly higher than that in the natural population. Moreover, the increase in the proportion of pollinated flowers could significantly increase seed set per flower, and pollinator visitation rate was the important limiting factor for seed set in both populations. *Andrena lebedevi* Popov was found to be the most frequent pollinator in both populations. Outcrossing was dominant in the breeding system and insect pollination played an important role in outcrossing. Our study suggested that proper management (artificial selection) could promote pollination success of *M. sativa*.

## 1 INTRODUCTION

Plants are immobile, and therefore, rely on abiotic or biotic vectors to transport pollen for sexual reproduction (Ashman et al., 2004). Pollination is a key biological process in terrestrial communities, and it affects a variety of evolutionary processes, such as selection on floral attraction and plant mating systems (Ashman & Morgan, 2004). For many plant species, pollination is the first stage in sexual reproduction, and an essential prerequisite for the development of fruits and seeds (Ashman et al., 2004; Kevan, Clark, & Thomas, 1990). The plant pollination process provides important indicators for designing conservation and sustainability strategies for a population (Rodríguez‐Oseguera, Casas, Herrerías‐Diego, & Pérez‐Negrón, 2013).

In recent years, plant–pollinator interaction has been the focus of much discussion and debate (Fenster, Armbruster, Willson, Dudash, & Thomson, 2004; Gómez, Abdelaziz, Lorite, Munõz‐Pajares, & Perfectti, 2010). Plant species that depend on animal pollinators for their reproduction have developed many different phenotypes traits, such as floral display, flower architecture and nectar (Arias‐Cóyotl, Stoner, & Casas, 2006; Nores, López, Rudall, Anton, & Galetto, 2013). Insect mediated pollination, the transfer of pollen within or between flowers via insect vectors, is one such ecosystem service that benefits human populations and agriculture (Fisher, Turner, & Morling, 2009). During this process, the quantity of pollen deposited on the stigma is limited by pollinator visitation frequency and affects pollination success rates (Ashman et al., 2004). Kishore, Shukla, Babu, Sarangi, and Patanayak (2012) also indicated the function of floral traits may not only to facilitate pollination by the primary pollinator but also to restrict other potential pollinators.

Pollinator visits are likely to be less frequent in stressful habitats (Neto, 2013). More specifically, it has been demonstrated that pollinators respond strongly to the local abundance of flowering plants, but habitat fragmentation may reduce the necessary resources to support resident pollinators (Kunin, 1997; Wagenius & Lyon, 2010). Fragmented habitats result in isolation and edge effects, and disrupting species interactions, such as plant–animal mutualisms. Steffan‐Dewenter, Klein, Gaebele, Alfert, and Tscharntke (2006) indicated pollinator abundance and activity decline with fragmented habitats owing to the reduction in floral rewards or habitats that could not meet the nesting requirements of pollinators. Similar pattern of pollinator activity was also documented between the wild and managed populations of *Myrtillocactus schenckii* (Cactaceae) (Fernando, Stoner, Pérez‐Negrón, & Casas, 2010). Recent studies have also shown that management may affect flowering patterns, pollinator foraging behavior, and pollination processes (Chen, Zhao, & Zuo, 2015; Quesada et al., 2004).

*Medicago sativa* is an important cash crop and valuable member of the plant community in the arid region of northwest China. *Medicago sativa* has great potential in terms of forage and medicinal uses (Jiang, Bi, He, & Zhang, 2003). Communities of this plant play a critical role in sand fixation and vegetation productivity. This study aimed to test the effect of human management on pollinator visits and seed set of *M. sativa*. Our specific objectives were to (a) examine how the management affect the number of flowers produced and the seed set per flower, (b) estimate the relative impact of pollinator visitation rates on pollination success throughout the proportion of visited flowers and pollinated flowers, and (c) determine how pollinators and pollinator activity influence seed set per flower. Furthermore, we assessed the relationships between pollinators and pollination success in different populations.

## 2 METHODS

### 2.1 Species

*Medicago sativa* is usually 0.3–1.0 m in height and mainly distributed throughout Gansu, Ningxia and western Inner Mongolia provinces in China. There are three species, one subspecies in China. *Medicago sativa* is a perennial plant that varies in racemes (inflorescences) per stem and open flowers per raceme, creating large variation in floral display size among plants (Pedersen, 1953). In the present study, we selected purple flowers of *M. sativa*, and this species has four petals and 10 stamens (Jiang et al., 2003). In addition, *M. sativa* have a tripping mechanism, and the flower remains open following tripping, with the stigma and anthers exposed. In order to collect pollen and nectar, pollinators must trip a flower to release the anthers and the stigma.

### 2.2 Study area and experimental layout

The study area was at the Urat Desert‐grassland in western Inner Mongolia of China (between 41°06′–41°25′N and 106°59′–107°05′E), and the annual mean rainfall is approximately 153.6 mm.

**Figure 1:**
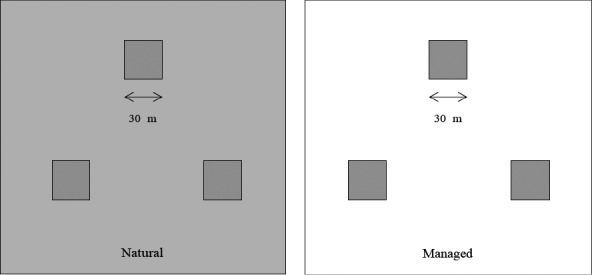
The experimental layout consisted of two studied patches and 6 plots in total, three natural plots (30 × 30 m) and three large managed plots (30 × 30 m). Natural plots were mirrored in a symmetrical arrangement and surrounded by undisturbed vegetation (gray area). In the managed patch, other species but *Medicago sativa* were cleared (white area). We remove vegetation and provide water and nutrient in the managed patch

This study was carried out from April 2013 to October 2017. The experimental layout consisted of two studied patches and six plots in total, three natural plots (30 × 30 m) and three large managed plots (30 × 30 m). For this study, the studied plots occurred in an area that was originally dry, arid land. In the natural patch, plants survives grew naturally without any artificial management, and the average density of *M. sativa* was 15 individuals per 100 m^2^, and the two patches were separated by 100 m in the study area. In natural population, there were some general plants, such as *Reaumuria songarica* (Pall.) Maxim and *Salsola passerina* Bunge. Moreover, the flowering time of these species were no overlap with *M. sativa*. In the managed population, the average density of *M. sativa* was also 15 individuals per 100 m^2^, and other species but *M. sativa* were cleared (five times per year). We also remove vegetation and provide water and nutrient in the managed patch. The managed population is nearly 300 m away from the natural plots, which had similar landforms to the natural population (Figure 1).

### 2.3 Phenology

Ten individual *M. sativa* plants per population were labeled and observed for phenological characters. The flower buds, flowers in anthesis and fruits on each branch were counted throughout the entire reproductive season (from April to September) for each plant. We then calculated the proportion of flower buds, flowers in anthesis and mature fruits.

### 2.4 Floral traits

We marked 20 plants per population while they were still budding to facilitate the assessment of the flowers when they were completely open. Moreover, the timing of the following events was also recorded. Video filming was conducted continually throughout anthesis in 3–4 flowers in marked plants. In addition, we measured the structure of inflorescences, the length of flower (from petal tip to flower base), calyx and flower stalk.

### 2.5 Managed experiment

In study area, we marked 36 flowering plants (18 natural plants and 18 managed individuals). In each plot, the six labeled plants were at the same flowering stage. Three treatments were set up to estimate the relative impact of managed experiment on flower number and seed set in different populations: (a) Control, plants experienced their natural environment; (b) Water added, plants had 60% more water than the control through weekly watering in the flowering period; (c) Fertilizer added, a liquid nitrogen–phosphorus–potassium fertilizer (N:P:K, 9:2:6) was applied around the base of the plants once a month during the flowering season (1% v: v dilution, 20 ml per plant) (Rowan et al., 2008). We collected the flower number and seeds set per flower produced by the control, water, and fertilizer treatments. Moreover, we counted the number of seeds and ovules per flower according to the following equation:

$$\text{Seed set}=\frac{\text{Number of seeds per flower}}{\text{Number of ovules per flower}}\times 100\%$$

### 2.6 Pollinator observations

To determine the identities and quantities of pollinators, we conducted surveys of pollinators from May to July. In each population.

We labeled 20 racemes (10 flowers per raceme) and repeatedly observed throughout the complete process of anthesis. We used fixed video cameras to assess the duration of each pollinator visit, including pollinators that were collecting pollen and nectar. We carefully analyzed the presence or absence of pollen grains adhering to the bodies of the pollinators and determined whether they contacted stamens and stigmas. In addition, images of the pollinators were used for identification in the laboratory. The flower visitation frequency of pollinators was calculated according to the following equation (Goverde, Schweizer, Baur, & Erhardt, 2002):

$$\text{Visitation frequency}=\frac{\text{Number of visits}}{\text{Number of flowers}\cdot \text{Observation time}}$$

### 2.7 Effect of pollinator visitation rate on pollination

We marked 18 flowering plants (nine natural and nine managed plants) while they were still budding. For each plant, 20 flower buds were randomly chosen and marked with tags. We noted the flowering stages and growth progress of the marked flowers throughout video filming.

To determine how pollinator visitation rates affected pollination success, we investigated the proportions of flowers anthesis, pollinator visitation, pollination, and seed production at 2‐week intervals from May to September when all seeds were mature. Fruits produced were collected in early August and the length of fruits (pots) and the number of seed were examined in the laboratory. Ovary enlargement was a valid criterion for assessing fertilization of ovules (Garwood & Horvitz, 1985; Suzuki, 2000). In these bagged and not pollinated flowers, we measured the length of ovaries in these un‐pollinated flowers when other flowers were fruiting. The average length of ovaries was 5 ± 0.39 mm (*n* = 20, Mean ± *SE*). Flowers with ovaries (pots) longer than 5 mm were classified as successfully pollinated. Conversely, even if flowers were tripped open, these flowers in which the length of ovary was less than 5 mm were classified as visited by pollinators but not as successfully pollinated. The percent of fresh flowers and mature seeds were recorded and calculated according to the following equation (Suzuki, 2000):

$$\text{Percentage of pollinated among visited flowers}=\frac{P}{V}\times 100\%$$

$$\text{Percentage of seeds among visited flowers}=\frac{S}{V}\times 100\%$$

$$\text{Percentage of seeds among pollinated flowers}=\frac{S}{P}\times 100\%$$

where *P* is the proportion of pollinated flowers, *V* is the proportion of visited flowers, and *S* is the proportion of seeding per flower on marked plants.

### 2.8 Breeding system

The breeding system experiments were analyzed using samples of 20 individual plants from both natural and managed populations. Six experimental treatments were conducted prior to anthesis, in which we sampled 360 flowers (10 flowers per raceme, six racemes per plant) in each population. Each treatment has 60 flowers, and these flowers were on the same plant. Experimental treatments were as follows:

Natural pollination (control): the flowers were marked and maintained under natural conditions.
Manual cross‐pollination: samples of flowers received additional pollen (hand‐pollination) collected from individual plants more than 20 m away, and flowers that received the pollen were emasculated before pollen liberation.
Nonmanipulated cross‐pollination: the flowers were without any manipulation, and these flowers were also emasculated.
Manual self‐pollination: flowers were pollinated with their own pollen and then bagged to prevent insects and wind pollination.
Nonmanipulated self‐pollination: the inflorescences were bagged without any manipulation of the flowers.
Emasculation and netting: the stamens of the flowers were removed before pollen liberation, and nets of 1 mm^2^ mesh were used to prevent insect pollination.

In October, we counted the number of seeds and ovules per flower for each treatment.

### 2.9 Self‐compatibility Index

We calculated the self‐compatibility index (SCI) according to the following equation:

$$\text{Self‐compatibility index}=\frac{\text{The seed set of self‐pollination}}{\text{The seed set of manual cross‐pollination}}$$

Self‐compatibility index values of ≤0.2 indicate self‐incompatibility, whereas values >0.2 show self‐compatibility (Zapata & Arroyo, 1978).

### 2.10 Data analyses

A general linear model was used to determine the effects of different populations (natural and managed) and treatments (control, water, and fertilizer) on flowers and seed set. The model used different populations and treatments as fixed factors, the proportion of flower number and seed set as the dependent variable. We also used a general linear model to determine the effects of pollination treatments and different populations on seed set. We used pollination treatments and different populations as fixed factors, and seed set as the dependent variable in the model.

We tested whether pollinator visitation rates increased pollination success. We performed regression analyses using the statistical software package SPSS 19.0, modeling the proportion of pollinated flowers to female reproductive success (calculated the proportion of seeding per flower on marked plant). The model used the proportion of pollinated flowers as the independent variable, and the proportion of seeding per flower as dependent variable.

We used ANOVA to compare the production of flowers in anthesis and mature fruits between natural and managed populations. All analyses were performed using the statistical software package SPSS 19.0 for Windows (SPSS Inc., Chicago, IL, USA).

## 3 RESULTS

### 3.1 Phenology

**Figure 2:**
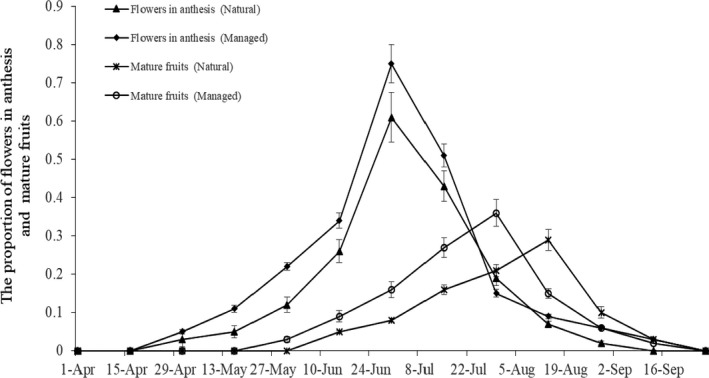
Phenology of *Medicago sativa* in natural and managed populations. Proportion of reproductive structures: flowers in anthesis and average number of mature fruits. Average number ± *SD* of phenological state structures per individual of *M. sativa* throughout the reproductive season

In both populations, flowering of *M. sativa* typically occurs from late May until late July. However, the period of peak flowering was different between the natural and managed populations, with the latter experiencing a longer flowering period (Figure 2).

Our results indicated that mature fruits were available from June to September, though fruit production peaked at different times among the populations. The natural population reached its peak at mid‐August, while the managed population peaked during the last week of July (Figure 2). In addition, the average number of mature fruits per branch in the managed population was significantly higher than that in the natural population (*F* = 15.11; *p* < 0.05).

### 3.2 Floral traits

Our observations indicated flowers of *M. sativa* have predominantly diurnal anthesis. In the natural population, flowers opened at approximately 08:30 and began to close at approximately 14:00. The flowers of managed population began opening at approximately 08:00 and were completely open by approximately 08:30. In general, anthesis started earlier in those flowers receiving solar radiation first. In addition, the flowering period of a single flower (from the time of petal opening to the stamen and petal wilting point) was 6 days in the managed and 5 days in the natural population.

Our results indicate that the average density of individuals was similar in both populations, but the number of flowers produced per branch in the managed was significantly higher than that in the natural population (*F* = 10.54, *p* < 0.05). Moreover, the total flower availability per plant was also significantly different in the natural and managed populations.

We observed 60 flowers in marked plants, *M. sativa* usually has fascicled racemes, and there are 5–30 flowers per raceme, 1–5 raceme per stem. The length of flower, calyx and flower stalk in mm was (Mean ± *SD*): 8.69 ± 2.16, 3.87 ± 1.12 and 1.86 ± 0.61, respectively.

### 3.3 Managed experiment

**Figure 3:**
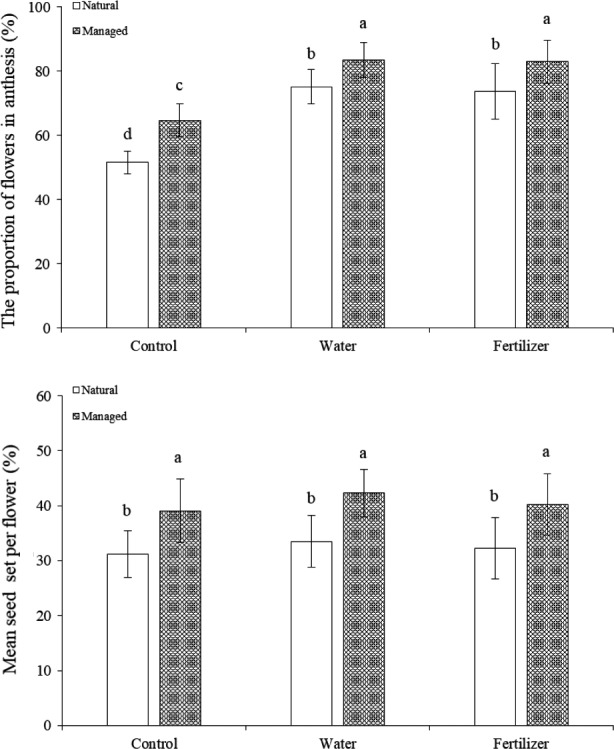
The proportion of flowers in anthesis and mean seed set per flower under managed experiment in both populations

In natural population, our results indicated that water‐added increased the proportion of flowers in anthesis and differed significantly between the water‐added and control (*F* = 38.26, *p* < 0.05). In addition, the proportion of flowers in anthesis was similar between the water‐ and fertilizer‐added (*F* = 0.20, *p* > 0.05; Figure 3). We also found strong effects of both water‐ and fertilizer‐added on the proportion of flowers in anthesis in the both populations.

In the natural population, the seed set per flower did not differ significantly between the control and fertilizer‐added flowers, at 31.2 ± 4.3% in the control and 33.5 ± 5.6% in the fertilizer‐added flowers. The seed set per flower also did not significantly differ between the control and the water‐added flowers (32.3 ± 4.7%; *F* = 1.21, *p* > 0.05). Moreover, the control, water‐ and fertilizer‐added plants produced similar the mean seed set per flower in the managed plots, indicating that water‐ and fertilizer‐added had no detectable effect on the mean seed set per flower.

### 3.4 Pollinators and pollinator activity

In the studied patches, *M. sativa* were visited by *Andrena lebedevi* Popov, *Eutricharaea manchuriana* Yasumatsu, *Megachile spissula* Cockerell, *Apis mellifera* Ligustica Spinola and *Xanthosaurus remota* Smith. The flowers of *M. sativa* have a tripping mechanism. Pollinator activity acts as a tripping agent, and pollinators do so by depressing the keel petals at the base of the flower.

Moreover, pollinators left flowers, the stigma of tripped flowers pressed against the banner petal and can't retract to its original position. We were able to identify flowers whether had been visited or pollinated. *Andrena lebedevi* was the first species to visit the flowers; its activity started at 08:00 and finished at 18:00. *Andrena lebedevi* landed on the front of the flower and used its head to push the flap forward while using the forefoot and hind foot together to trip the flap. Its mouthpart entered the base of the petals to collect nectar and pollen. This action may be explained by the large and hairy bodies of this species, which can easily carry and deposit more pollen.

**Figure 4:**
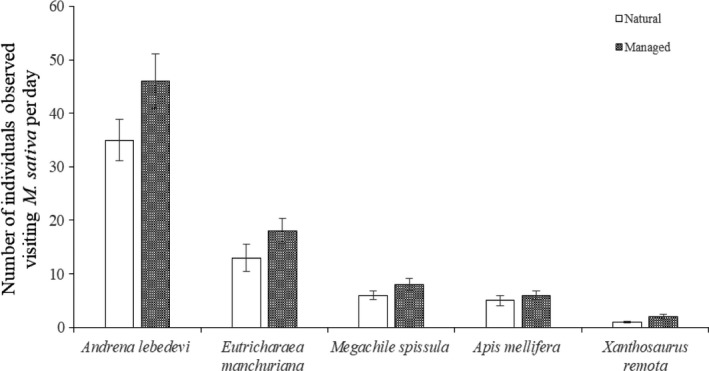
Number of individuals observed visiting *Medicago sativa* per day in two studied patches

**Figure 5:**
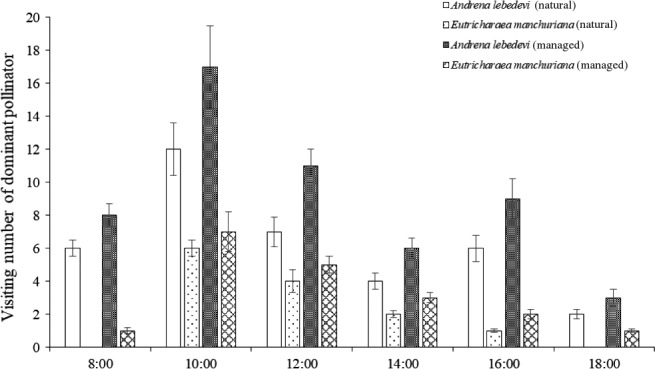
The visiting number of dominant pollinator per day to *M. sativa* in natural and managed populations

In the natural population, the visiting pattern of 235 pollinators was observed. Among them, 138 pollinators belonged to *A. lebedevi*. Pollinators visited these flowers from while they were open and carried away nectar and pollen. In the managed population, 317 pollinators were recorded of which 196 pollinators were *A. lebedevi*. Number of pollinators per day in the managed population was significantly higher than that in the natural population (*p* < 0.05; Figure 4). The flower opening and pollen release occurred between 08:00 and 15:00, and this period coincided with the most frequent activity of *A. lebedevi* (Figure 5). Our results showed *A. lebedevi* was the most effective and frequent pollinator between the natural and managed populations.

### 3.5 Pollinator visitation rate

**Figure 6:**
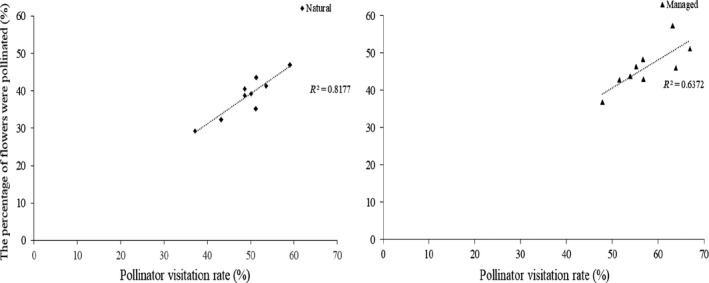
The relationship between the proportion of pollinated flowers and the pollinator visitation rate for *Medicago sativa*

**Figure 7:**
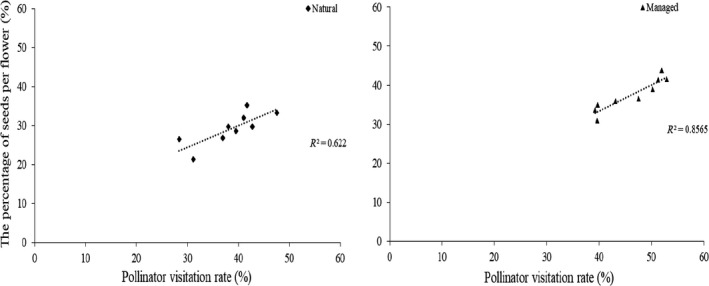
The relationship between the proportion of seeds per flower and the pollinator visitation rate for *Medicago sativa*

In the natural population, 49.21% of flowers were visited (*V*) at least once by effective pollinators, and 38.56% of flowers were pollinated (*P*). The pollination percent among visited natural flowers (*P*/*V* × 100%) was 78.36%. In addition, 29.23% of flowers produced seeds (*S*), resulting in the percent of seeds among visited flowers (*S*/*V* × 100%) and pollinated flowers (*S*/*P* × 100%) to be 59.40% and 75.80%, respectively. In the managed population, our outcomes showed that 57.35% of flowers were visited, 46.17% of flowers were pollinated and 37.51% of flowers produced seeds. We found the percent of seeds among pollinated flowers to be 81.24%. These results showed that the percent of seeds among visited flowers and among pollinated flowers were significantly correlated with pollinator visitation rates in the natural population (the percentage of pollinated flowers: *r* = 0.90, *p* < 0.05; the percentage of seeds: *r* = 0.78, *p* < 0.05) and the managed population (the percentage of pollinated flowers: *r* = 0.79, *p* < 0.05; the percentage of seeds: *r* = 0.93, *p* < 0.05; Figures 6 and 7). These outcomes showed that higher pollinator visitation rates resulted in a higher percentage of seeds. In both populations, our results indicated that the pollinator visitation rate (49.21% in the natural and 57.35% in the managed) was the important limiting factor for seed set.

### 3.6 Breeding systems and self‐compatibility index

**Figure 8:**
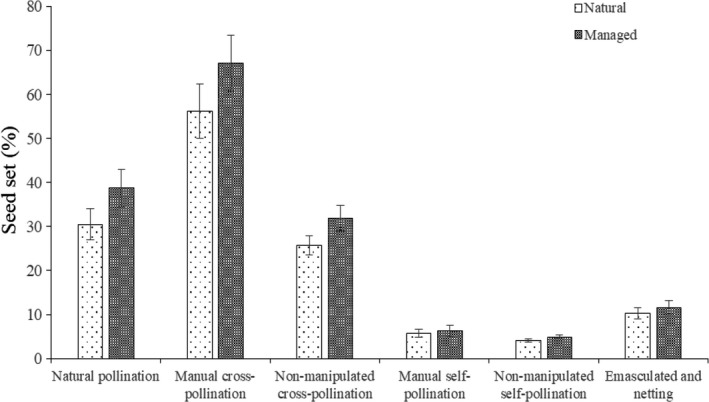
Seed set of *Medicago sativa* in different treatments

The seed set obtained in each pollination treatment is shown in Figure 8. The seed set of manual cross‐pollination was 56.2 ± 6.1% in the natural population, and 67.1 ± 6.3% in the managed population. We found the seed set of the managed population was significantly higher than that of the natural population (*p* < 0.05). In addition, our results indicated that the natural pollination seed set was 30.5 ± 3.5% for the natural population and 38.7 ± 4.3% for the managed population. In both populations, the seed set of manual cross‐pollination was significantly higher compared with the seed set of natural pollination (natural and managed population: *p* < 0.01). We found that outcrossing was dominant in the breeding system of *M. sativa*.

The nonmanipulated self‐pollination treatment resulted in 4.1 ± 0.4% seed set in the natural population, and 4.9 ± 0.5% in the managed population. Under the emasculation and netting treatment, the seed set was only 10.3 ± 1.2% for the natural population and 11.6 ± 1.5% for the managed population. In both populations, the seed set of nonmanipulated cross‐pollination was significantly greater than that of the emasculation and netting treatment (natural and managed population: *p* < 0.01). These results indicated that insect pollination played more important role than wind pollination in the outcrossing system.

In the natural population, 5.7% of the seed set under manual self‐pollination and 56.2% in the manually cross‐pollinated set. The SCI value was 0.10, which indicated this species was self‐incompatible.

## 4 DISCUSSION

### 4.1 Effect of floral traits and pollinator behavior on seed set

Floral traits might have evolved in order to attract and utilize more groups of pollinators in most flowering plants (Fenster et al., 2004). Sandring and Agren (2009) have pointed out that the evolution of floral display is commonly attributed to pollinator‐mediated selection in most animal‐pollinated plants. Kishore et al. (2012) indicated the function of floral traits may not only to facilitate pollination by the primary pollinator but also to restrict other potential pollinators, and these traits could be better transferred pollen. In this study, the period of peak flowering of *M. sativa* were longer in the managed than that in the natural population. Moreover, the flowering peak is the highest offer of pollen and increases the possibility of insect pollination at this moment in *M. sativa*. In many species, floral differences between both populations have been associated with environmental heterogeneity, which would be magnified by human disturbance (Chen et al., 2015). The difference of management suggested that water‐ and fertilizer‐added could affect the number of flowers produced, but had no detectable effect on the mean seed set per flower.

Many studies suggest that the tripping mechanism to some extent improves the efficiency of insect pollination (Gopinathan & Babu, 1987; Liu, Li, & Wang, 2008; Strickler & Vinson, 2000). In *M. sativa*, the stigma and style emerge and protrude from the keel, and pollinators usually visit the lower side of the petals. When the insects leave the flower, the stigma of tripped flowers pressed against the banner petal, providing a signal to pollinators that the flower has been visited and increase the efficiency of insect pollination. Thus, the tripping mechanism of *M. sativa* allows these flowers to achieve efficient insect pollination, and outcrossing also favors the production of a diverse population. Moreover, Strickler and Freitas (1999) also indicated *M. sativa* flowers wilt within hours of being tripped, but un‐tripped flowers can remain open for a week. This traits resulted in the rate of pollinator visited and tripping increases, the rate at which plant increasingly puts resources into developing seeds. This also explains why seed set in the managed population were higher than that in the natural population.

### 4.2 Pollinator visitation rates limit pollination success

Pollination success is related to pollinator type, as different pollinators vary in pollination effectiveness (Gómez, Bosch, Perfectti, Fernández, & Abdelaziz, 2007). Goverde et al. (2002) reported that a managed environment could affect pollinator diversity and abundance in the natural population. In our study, the single flowering period in the managed flowers was longer than that in the natural flowers. Moreover, the total flower availability per day was significantly higher in the managed population than that in the natural population, which could explain the difference of pollinator visitation rates between both populations.

Many researchers have studied the relationships between pollinator visitation rates and plant reproduction, but how pollinator visitation rates affect plant reproductive success still need further study (Bauer, Clayton, & Brunet, 2017; Sletvold & Agren, 2010; Stephenson, 1981; Zimmerman & Pyke, 1988). In present study, we were able to identify whether flowers had been visited by effective pollinators because of the tripping mechanism of *M. sativa*. A similar pattern of pollinator visitation also documented in *Cytisus scoparius* (Leguminosae), reaffirming the effect of pollinator visitation rates on plant reproduction (Suzuki, 2000). Bauer et al. (2017) suggest that the increase in the proportion of pollinated flowers combined significantly increased seed set per raceme. Our outcomes also support this view that both the regressions between the proportion of pollinated flowers and seed set per flower was statistically significant, indicating that the increase in the proportion of pollinated flowers could significantly increase seed set per flower and the visitation rate is an important limiting factor for pollination success.

### 4.3 Pollinator activity and breeding system in different populations

Pollination by animals is largely considered a co‐adaptive process in which plants evolve traits to attract certain pollinators, whereby pollinators improve the efficiency of their activities to better exploit the floral resources of plants (Sharma, Shaanker, Leather, Vasudeva, & Shivanna, 2011; Sunnichan, Mohan Ram, & Shivanna, 2004). In *M. sativa*, the period between 08:00 and 12:00 appeared to be crucial for pollination of most flowers since this was when the flowers had the largest opening and there was overlap in pollen release. This period coincided with the most active time for *A. lebedevi*. When *A. lebedevi* contacted the anthers, a large amount of pollen was collected on its legs and because of its suitable body size. The visitation activity of *A. lebedevi* in the managed population was more frequent than that in the natural population likely because pollinators preferred to visit areas with greater resource availability. Therefore, the pollination success of *M. sativa* in the managed population was higher than in the natural population.

In many species, plant fitness may be lower if pollinator activities are missing or reduced (Pavlik, Ferguson, & Nelson, 1993). Previous studies indicated that reduced pollinator activity could disrupt plant‐pollinator interactions, and thus reduce seed set and gene flow of plant populations (Jennersten, 1988; Robertson, Kelly, Ladley, & Sparrow, 1999). Empirical evidence suggests that changes in pollinator activity might strongly influence plant pollination success (Goverde et al., 2002). Our results showed *M. sativa* is self‐incompatible, and insect pollination played an important role in outcrossing. Many researchers have pointed out that floral display size is a good indicator of reward, it is common that pollinators selection on floral display size (Bauer et al., 2017; Worley & Barrett, 2000). The environment may affect floral display size, and it also plays an important role in pollinator activity for a reward. In the managed population, plants had greater resource availability to attract more pollinators because human management provided water and mown. This may support the assertion that environmental heterogeneity affects pollinator activity and pollination success.

## CONFLICT OF INTERESTS

All authors declare that we do not have any competing financial interests.

## AUTHOR CONTRIBUTIONS

MC designed the experiment. MC wrote the manuscript; XYZ and XAZ provided editorial advice. All the authors have read and approved the manuscript. We thank Urat Desert‐grassland Research Station and Naiman Desertification Research Station for all the help and support during this study.
